# Severe Craniofacial Trauma After Multiple Pistol Shots

**DOI:** 10.1515/med-2019-0072

**Published:** 2019-08-31

**Authors:** Ingrid Haller, Wolfgang Lederer, Bernhard Glodny, Franz J. Wiedermann

**Affiliations:** 1 Department of Anaesthesiology and Critical Care Medicine, Medical University of Innsbruck, 6020 Innsbruck, Austria; 2 Department of Radiology, Medical University of Innsbruck, 6020 Innsbruck, Austria

**Keywords:** Pistol shot, Head injury, Airway management

## Abstract

A 48-year-old woman suffered from life-threatening injuries in head and chest caused by six pistol shots fired at close range in an attempted homicide. We report here on our successful airway management and bleeding control at the scene of crime and the multidisciplinary surgical treatment of the associated head and neurovascular injuries.

## 1 Introduction

Severe craniofacial trauma is frequently associated with severe hemorrhage and impaired breathing especially when the lower face is injured [1]. In addition, there is a risk of associated injuries to the cervical spine, base of the skull, carotid arteries and the brain [2]. Orotracheal intubation and packing was reported to be sufficient primary management with about half of the patients needing further surgical repair [3]. Emergency intubation in civilian penetrating gunshot injuries was reported in 7 to 13% of cases, the need for an early surgical airway was between 11% and 35% [1, 2, 3, 4]. Impaired airways were more frequently observed after high-velocity injuries and in cases with injuries to the tongue, floor of the mouth, and midline or bilateral facial skeletal bones [4]. Maintaining airway and oxygenation and supporting circulation are crucial in treatment of victims at the scene of crime [5], particularly when mask ventilation and supraglottic airway devices are impractical and intubation difficult [6]. Whenever ventilation fails, at least lateral positioning of the patient should be established to achieve airway patency and drainage of blood [7]. As the outcome mainly depends on airway management and severity of associated head or neurovascular injuries requiring immediate intervention, patients should be transported to a trauma center equipped to deal with craniofacial and neurosurgery [1].

## 2 Case report

**Figure 1 a-d:**
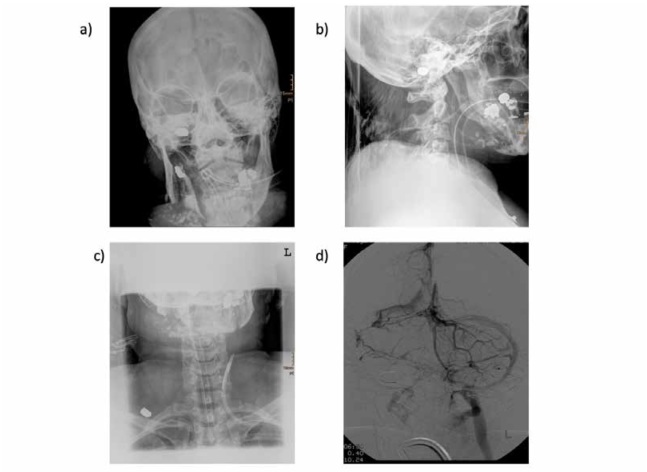
Smashed mandibular bone, with metal stemming from teeth restorations, and from the projectiles. Intact projectiles are seen in the sigmoid sinus (a and b), and the soft parts of the dorsum subcutaneously on the right side (c). The transverse and sigmoid sinus, and the jugular vein on the right side are blocked (d; venous phase after right vertebral artery injection).

**Figure 2 a-d:**
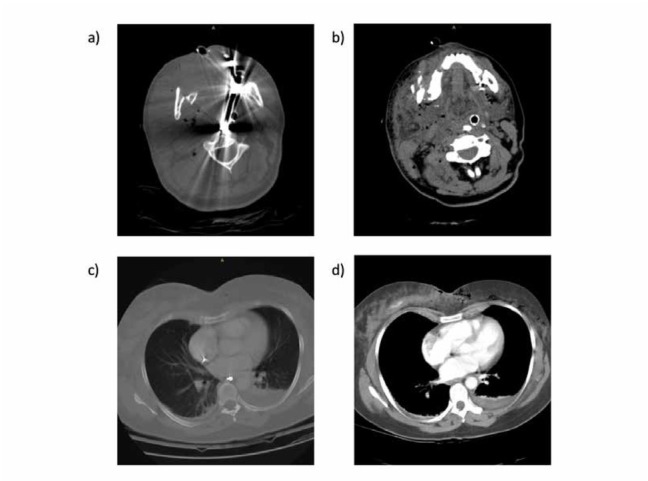
Mandibular fragments, teeth and teeth restoration fragments as well as the respiration tube in the perimandibular region, and the completely swollen pharynx (a and b). Teeth fragments in the esophagus, as well as a small pleural effusion (c), and active extravasation in a projectile trajectory within the right mamma (d).

**Figure 3 a-b:**
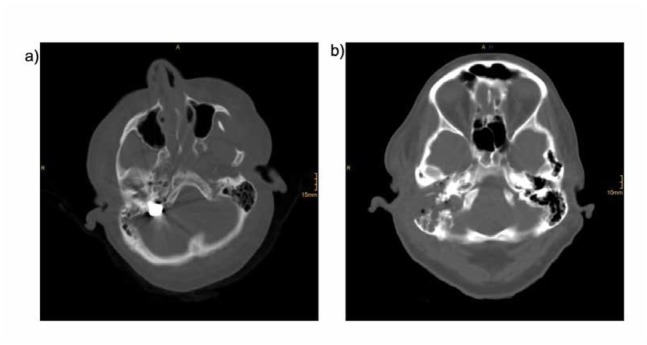
Axial computed tomography image of the base of the skull showing the bullet within the distal part of the sigmoidal sinus on the right side (a). A bony defect is seen after transmastoidal removal of the bullet (b).

A 48-year-old woman suffered from life-threatening injuries to head and chest caused by six pistol shots fired at close range in an attempted homicide. When the emergency physician arrived on scene, the patient presented with oral bleeding from a smashed mandible (Figure 1) and with lacerated tongue causing mechanical obstruction of the airway. Rapid sequence induction was performed with 3 mg midazolam and 50 mg S-ketamine. Despite pharyngeal suction, visualizing the vocal cords with conventional direct laryngoscopy was impossible. The patient had spontaneous breathing and the tracheal tube then was inserted where air bubbles arose from the bleeding tissue. Proper position was confirmed by capnography and volume-controlled mechanical ventilation was started. Pharyngeal packing with gauze was performed to achieve hemostasis. Crystalloids were administered, and anesthesia was maintained with 0.2 mg fentanyl and 10 mg midazolam. In the emergency department, tracheostomy was established and mechanical ventilation was continued by pressure-controlled ventilation. After clinical and radiologic evaluation, under general anesthesia conservative debridement of all devitalized tissues was performed by a multidisciplinary team comprising an ear, nose and throat (ENT) surgeon and a craniofacial surgeon. Following the trajectories of the missiles, one bullet was removed from the lower jaw (Figure 1, 2) and mandibular osteosynthesis was performed via buccal approach using miniature plates and screws [8]. Ten units of packed red cells were administered during the operation. The patient was transferred to the intensive care unit (ICU) and pressure support ventilation was continued for six days. Repeated chest x rays revealed minimal mediastinal hematoma and mild pleural effusion on the left side but no signs of pulmonary infection. Ten days later, with an approach via the right mastoid process, the ENT surgeon removed the planum of the mastoid with the help of an 8 mm driller, the mastoid showed multiple fractures and was filled with blood. Extended mastoidectomy was carried out. The inferior part of the mastoid had been destroyed by the bullet that had entered the posterior skull base and was removed with the help of a hook. The dura and the sinus had been destroyed in this part, and a 5-cm-long thrombus was removed from the right transverse sinus. The surgeon also repaired the dural tear, and the neurosurgeon established a lumbar decompressive drainage. The facialis nerve was not interrupted but partly the nerve fibers were exposed. Even though there were hematomas and massive swelling in the extratemporal course of the facial nerve, nerve continuity had been preserved so that there was a good chance of spontaneous regeneration. Thus, there was no indication for an interposition nerve graft for the facial nerve (Figure 3). The patient recovered and could be discharged from ICU on day 14. She survived without neurological damage except for Bell’s palsy on the right side. The patient underwent several reconstructive interventions by the plastic surgeon including cross-face operations for residual paresis of the facial nerve.

## 3 Discussion

We performed a retrospective evaluation of initial injury and emergency treatment, surgical repair and course of recovery and outcome in a patient who suffered penetrating craniofacial and chest injuries by six pistol shots fired at close range. Severe craniofacial trauma from bullet wounds has a high potential for fatal outcome [5]. Treatment plan depends on associated vascular injuries, intracranial communication of lesions, and injuries to any other adjacent vital structures. Establishing a secure airway is of paramount importance [6]. In the case presented here, rapid sequence induction was performed with moderate doses of midazolam and S-ketamine but without administration of any muscle relaxant to maintain spontaneous breathing. Fortunately, tracheal intubation was established under guidance of air bubbles from the bleeding tissue after direct laryngoscopy had failed. However, emergency physicians should be trained and be prepared to establish a surgical airway by cricothyroidotomy on the scene when conventional measures fail. Our patient had suffered injuries to the lower face and the mandible that caused severe hemorrhage and impaired breathing [1]. Gunshot wounds to the mandible are common [9]. Some gunshot wounds are through-and-through injuries, but in many patients, as in our case, bullets may enter with no visible exit wound [9]. Most facial wounds can be treated by craniofacial surgeons in a conservative manner immediately after the injury with good functional and cosmetic results [10]. Soft tissues can be repaired by primary closure or local flaps, but distant flaps are more likely to be performed as a secondary procedure [9]. Infections are not frequently observed and rates do not differ between patients undergoing early and late reconstructions [11]. In the case of our patient, the gunshot wound presented with signs of facial nerve paralysis and cerebrospinal fluid (CSF) leak. Generally, surgical removal of intracranial bullets is highly controversial, but CSF fistulae are a potential risk for infection [12]. During decompression of the facial nerve, and vascular and dural repair in the close vicinity of the bullet, the ENT surgeon successfully removed the missile without further injury to the tissue.

## 4 Conclusion

Immediate airway management and bleeding control followed by coordinated multi-disciplinary treatment by the emergency physician, anesthesiologist, intensivist, radiologist, ENT surgeon, craniofacial surgeon, neurosurgeon and plastic surgeon is crucial for the successful treatment of penetrating craniofacial trauma [6,7].
